# Books and Magazines

**Published:** 1907-03

**Authors:** 


### Books and Magazines.

STARR ON NERVOUS DISEASES.—Organic and Functional Nervous Diseases. By M. Allen Starr, M. D., Ph. D., LL. D., Professor of Neurology in the College of Physicians and Surgeons, New York; ex-President of the American Neurological Association and of the New York Neurological Society. Second edition, thoroughly revised. Octavo, 824 pages, with 282 engravings and 26 full-page plates. Cloth, $6, net; leather, $7, net. Lea Bros. & Co., Philadelphia and New York, 1907.

The author's position in the forefront of neurologists has been shown anew in the rapid exhaustion of the first edition of his work, limited though it was to organic nervous disease. An even warmer reception is assured for this revision, which brings the organic portion to date and adds a section covering the functional diseases, so that the volume now presents the whole field of neurology as understood and practised by a master. The author is the reverse of abstruse or nihilistic. On the contrary, he is straightforward and direct, and justifies his optimism as to the advanced position of neurological diagnosis and treatment by the wealth of information placed at command of his readers. Paying due regard to theory, he devotes especially full attention to etiology, diagnosis and treatment, both medical and surgical. The book is largely based on the solid foundation of long experience, but it also embodies the well-attested knowledge of other authorities as gleaned from a thorough sifting of the vast literature of neurology. Practical, authoritative, covering the whole subject in all its aspects, and abundantly illustrated, this new edition of Prof. Starr's work answers the needs of students, practitioners and specialists.

PRACTICAL MEDICINE.—Series 1906. Year Book Publishing Co., Chicago.

This series consists of ten volumes, covering the field of medicine in every department, gleaned from the medical journals of the year. In our last issue we noticed all the series except Volume X, which is just at hand. It is "Skin and Venereal Diseases, Nervous and Mental Diseases," by Baum Patrick Healey. Price per volume, $1.25. The set of ten for $10.

---

Practical Dietetics With Reference to Diet in Disease. By Alida Frances Pattee, Graduate Boston Normal School of Household Arts; Late Instructor in Dietetics, Bellevue Training School for Nurses, Bellevue Hospital, New York City; Special Lecturer at Bellevue, Mount Sinai, Hahnemann, and the Flower Hospital Training Schools for Nurses, New York City; St. Vincent de Paul Hospital, Brockville, Ontario, Canada. Fourth edition. 12mo, colth; 300 pages. Price, $1, net. By mail, $1.10. C. O. D., $1.25. A. F. Pattee, Publisher, 52 West 39th St., New York.

A text-book for the physician, student and nurse as a general guide for proper diet in disease. A work on the preparation of proper food for the sick and convalescent, giving in detail the method of preparing and administering liquid, semi-liquid and solid food. Contains the diet lists and what to avoid in various diseases; also the proper diet for infants and children as advised by leading physicians and hospitals of New York and Boston.

This book fulfills the requirements as to simplicity, brevity and exactness with reference to the dietetic treatment in disease and represenst the course in dietetics as arranged for and used at Bellevue Hospital.

This text-book has been in such demand that three large editions have been published since July, 1903.

Added to the authorized text-book list of the New York public schools, listed No. 3807.

Adopted by the United States government, Medical Department of the Army.

Adopted by the Canadian government, permanent schools of instruction for the Canadian militia.

---

The Practitioners' Visiting List for 1907. An invaluable pocket-sized book containing memoranda and data important for every physician, and ruled blanks for recording every detail of practice. The Weekly, Monthly and Thirty-Patient Perpetual contain 32 pages of data and 160 pages of classified blanks. The 60-Patient Perpetual consists of 256 pages of blanks alone. Each in one wallet-shaped book, bound in flexible leather, with flap and pocket, pencil and rubber, and calendar for two years. Price by mail, postpaid, to any address, $1.25. Thumb-letter index, 25 cents extra. Descriptive circular showing the several styles sent on request. Lea Brothers & Co., Publishers, Philadelphia and New York, 1906.

This well-known and popular book has been thoroughly revised and brought up-to-date.

Peterson's Obstetrics.—The Practice of Obstetrics. By Eminent Authorities. Edited by Reuben Peterson, A. B., M. D., Professor of Obstetrics and Diseases of Women in the University of Michigan, Department of Medicine and Surgery, Ann Arbor, Mich. Large octavo, about 1087 pages, with 523 engravings and 30 full-page plates in colors and monochrome. Cloth, $6, net; leather, $7, net; half morocco, $8, net. Lea Brothers & Co., Philadelphia and New York, 1907.

With the volume edited by Prof. Reuben Peterson, *The Practitioner's Library of Gynecology, Obstetrics and Pediatrics* is completed. It is fully up to the high standard set by its companions, the *Gynecology,* edited by Bovée, and the *Pediatrics,* by Carr. The profession now has at command in convenient form an authoritative exposition of the latest and best knowledge upon three closely interrelated and important specialties. The basic subjects of applied pathology and etiology are considered with sufficient fulness to lay the foundation necessary for a fruitful understanding of the practical aspects, to which major space is devoted. Each author has woven in his own observations of disease and the therapeutic measures which have resulted in the greatest success. This adds to each chapter a personal element of obvious value. In view of their particular importance in obstetrics, the series of illustrations has been made exceptionally rich, and it is likewise notable for being largely from original photographs taken from life. The facilities at Dr. Peterson's command have rendered it possible to an unusual degree to make selections specifically representing points in the text. Though it is to the interest of every practitioner to have this well-rounded and compact library at hand, the volumes are sold either together or separately.

---

PLASTER OF PARIS AND HOW TO USE IT. By Martin W. Ware, M. D., Adjunct Attending Surgeon, Mount Sinai Hospital; Surgeon to the Good Samaritan Dispensary; Instructor in Surgery, N. Y. Post-Graduate Medical School. 12mo; 72 illustrations, about 100 pages. Surgery Publishing Co., 92 Williams St., N. Y. City. Cloth, $1.

This is one of the most useful books ever presented, not only on account of the general demand for the information and instructions upon the subject which this book so explicitly, practically and comprehensively covers, but because this knowledge was not previously available except from such a vast experience as enjoyed by Dr. Ware, or, in part, by reference to many books on allied subjects.

It is a vivid narrative, profusely illustrated, of the many uses to which Plaster of Paris is adaptable in Surgery. The whole subject, from the making of the bandage to its use as a support in every form of splint, corset or dressing, is graphically described and illustrated. The use of Plaster of Paris in Dental Surgery is also covered. The book is presented in the artistic manner characteristic of the productions of the Surgery Publishing Company. It is printed upon coated paper and attractively bound in heavy red buckrum, stamped in white leaf and gold. Price, $1.

